# UDP-Glycosyltransferase Genes in the Striped Rice Stem Borer, *Chilo suppressalis* (Walker), and Their Contribution to Chlorantraniliprole Resistance

**DOI:** 10.3390/ijms20051064

**Published:** 2019-03-01

**Authors:** Jun Zhao, Lu Xu, Yang Sun, Pingping Song, Zhaojun Han

**Affiliations:** 1 Education Ministry Key Laboratory of Integrated Management of Crop Diseases and Pests, College of Plant Protection, Nanjing Agricultural University, Nanjing 210095, China; 2015202028@njau.edu.cn; 2 Institute of Plant Protection, Jiangsu Academy of Agricultural Sciences, Key Lab of Food Quality and Safety of Jiangsu Province-State Key Laboratory Breeding Base, Nanjing 210014, China; 3 Institute of Plant Protection, Jiangxi Academy of Agricultural Science, Nanchang 330200, China; sun2007yang@126.com; 4 Jiangsu Provincial Platform for Conservation and Utilization of Agricultural Germplasm, Institute of Botany, Jiangsu Province and Chinese Academy of Sciences, Nanjing 210014, China; songpingping@cnbg.net

**Keywords:** *Chilo suppressalis*, UDP-glycosyltransferase, chlorantraniliprole, resistance, RNAi

## Abstract

Uridine diphosphate glycosyltransferases (UGTs) are multifunctional detoxification enzymes, which are involved in metabolizing various chemicals and contribute to the development of insecticide resistance. However, the possible roles of UGTs in chlorantraniliprole resistance in *Chilo suppressalis* have rarely been studied in detail. Based on genome data, 24 UGT genes in *C. suppressalis* belonging to 11 families were identified, which were designated by the UGT nomenclature committee. Synergism assay data suggested that UGTs are potentially involved in chlorantraniliprole resistance in *C. suppressalis.* *CsUGT40AL1* and *CsUGT33AG3* were significantly overexpressed in the chlorantraniliprole resistant strain (12.36- and 5.34-fold, respectively). The two UGTs were highly expressed in the larval Malpighian tubules, fat body, and midgut; however, expression was lowest in the head. Injection of individual dsRNAs reduced the expression of the two target genes (by 69.34% and 48.74%, respectively) and caused significant higher larval mortality (81.33% and 54.67%, respectively). Overexpression of *CsUGT40AL1* and *CsUGT33AG3* was potentially involved in chlorantraniliprole resistance in *C. suppressalis,* as confirmed by the RNAi assay. Our findings suggest that overexpression of UGTs may contribute to chlorantraniliprole resistance in *C. suppressalis*.

## 1. Introduction

The striped rice stem borer, *Chilo suppressalis* (Walker), is an serious pest of agricultural grain crops, including rice, and it causes huge losses in yield throughout Asia [1,2]. Although it is known for its rapid development of resistance to many conventional insecticides, the management of *C. suppressalis* relies heavily on insecticides, which emphasizes the necessity for the understanding of its insecticide resistance mechanisms [3,4]. Chlorantraniliprole, as a novel diamide insecticide that targets the ryanodine receptor (RyR), has been extensively used for the control of caterpillars [5]. However, high-level chlorantraniliprole resistance of *C. suppressalis* has been reported after a few years of extensive use [6,7]. Understanding the chlorantraniliprole resistance mechanism can improve its application and prolong its use. Previous studies have found that three mutations (*G4910E*, *Y4667D*, and *I4758M*) in RyR might be involved in the chlorantraniliprole resistance in *C. suppressalis* [7,8]. Additionally, multiple overexpressed cytochrome P450-dependent monooxygenase genes (*CYP6CV5*, *CYP9A68*, *CYP321F3*, and *CYP32412*) potentially contributed to chlorantraniliprole resistance of *C. suppressalis* [9]. Enhancement of esterase activity might also confer *C. suppressalis* resistance to chlorantraniliprole [7,8]. These results showed complex resistance mechanisms in *C. suppressalis* to chlorantraniliprole. Therefore, the resistance mechanism of chlorantraniliprole in *C. suppressalis* should be clearly understood.

The insect detoxification systems that mediate diverse metabolic resistance mechanisms to insecticides are sophisticated, and different detoxification enzyme genes may be involved in insecticide resistance. In the three phases of detoxification, uridine diphosphate glycosyltransferases (UGTs) belong to phase II metabolizing enzymes that are found in all living organisms, which are encoded by a superfamily of genes [10]. The protein structure of UGTs contains two main parts: the aglycone substrate-binding domain at the N-terminus, and the UDP sugar donor-binding domain at the C-terminus. UGTs catalyze the transfer of glycosyl groups from a nucleotide sugar to a variety of small hydrophobic molecules (aglycones). This increases their water solubility and makes them easier to be excreted, resulting in the detoxification and elimination of substrates [11]. Therefore, glycosylation of toxins by UGTs is a particularly important detoxification mechanism. UGTs are important detoxification enzymes in insects which can metabolize insecticides, and consequently the involvement of insect UGTs in the development of insecticide resistance has been studied. Bull et al. (1972) found that UGTs could be involved in the metabolism of insecticides by enhancing glucosidic conjugation in the organophosphorus resistant *Heliothis virescens* [12]. Similarly, UGTs are important in pyraclofos metabolism in the resistant *Musca domestica* strain [13]. An important feature of detoxification enzyme–mediated metabolic resistance is the overexpression of detoxification enzyme genes at the transcriptional level in insecticide-resistant insects, which results in increased protein amounts and enzyme activity levels. Previous studies have suggested that gene overexpression is the cause of insecticide resistance. Overexpression of *UGT2* contributes to imidacloprid resistance in the resistant strain of *Leptinotarsa decemlineata* [14]. The overexpression of multiple UGT genes was found to play an important role in the resistance of *Aphis gossypii* to thiamethoxam insecticides [15]. Overexpressed *UGT2B17* in *Plutella xylostella* conferred high-level chlorantraniliprole resistance [16]. However, whether the chlorantraniliprole resistance in *C. suppressalis* is mediated by UGT genes needs to be investigated.

The study of the resistance mechanism in *C. suppressalis* will enhance the continued development of innovative alternative control measures and resistance management strategies. In the present study, UGT genes were identified in *C. suppressalis* based on genome data, and the phylogenetic relationships among these genes and their homologs in four other insects were analyzed. We performed synergism bioassays and screened overexpressed UGT genes using chlorantraniliprole-susceptible and chlorantraniliprole-resistant strains. Furthermore, we characterized tissue-specific expression profiles of overexpressed UGT genes. We also confirmed the function of UGTs in chlorantraniliprole resistance using RNA interference (RNAi). We aimed to elucidate the contribution of overexpressed UGTs to the chlorantraniliprole resistance mechanism in *C. suppressalis*.

## 2. Results

### 2.1. Chlorantraniliprole Toxicity and Synergists Assessment

The resistant strain (YYR) had developed a high resistance level to chlorantraniliprole (LC_50_ = 565.48 mg/L) with a resistance ratio (RR) of 44.32, while the susceptible strain (YYS) was sensitive to chlorantraniliprole. To investigate whether the UGTs were involved in the detoxification of chlorantraniliprole in the two strains of *C. suppressalis*, two chemicals, 5-nitrouracil (5-NU) and sulfinpyrazone (SUL), were selected as synergists for their good inhibition of UGT activity. In YYR, 5-NU increased the chlorantraniliprole toxicity by 3.38-fold, and SUL increased it by 3.56-fold. However, the toxicity of chlorantraniliprole was not significantly changed by 5-NU or SUL in YYS, and the synergistic ratios (SRs) were 1.22- and 1.13-fold, respectively (Table 1).

### 2.2. Identification and Classification of UGT Genes

**Figure 1:**
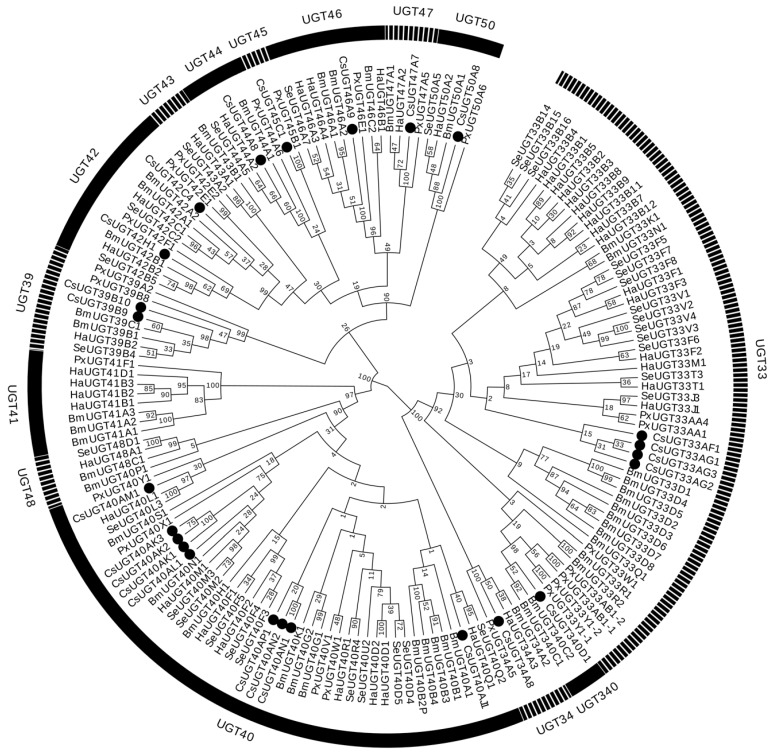
Phylogenetic relationship of UGTs from different species using the complete amino acid sequences. Twenty-four UGT amino acid sequences from *Chilo suppressalis* (Cs before the name), 23 from *Plutella xylostella* (Px), 32 from *Spodoptera exigua* (Se), 40 from *Helicoverpa armigera* (Ha), and 44 from *Bombyx mori* (Bm). The tree was constructed with MEGA 5.0, using the neighbor-joining method with the Poisson correction model and 1000 bootstrap replicates. The identified UGTs are highlighted with solid black circles.

Twenty-four UGT genes with complete open reading frames (ORFs) were identified from the genomic database of *C. suppressalis*, and their cDNAs were cloned using reverse transcription polymerase chain reaction (RT-PCR) confirmation. Amino acid sequences of 24 UGTs were predicted uridine diphosphate glycosyltransferase conserved domains ‘UDPGT’ based on SMART online software (http://smart.embl-heidelberg.de/). The UGT gene names were constituted by a prefix ‘*Cs*’ and a specific name. The specific names of these UGT genes were designated by the UGT Nomenclature Committee [17]. The characteristics of these 24 UGTs are summarized in Table 2. These gene sequences were deposited into GenBank (GenBank accession nos. MK135471-MK135494). The ORFs of the UGTs ranged from 514 to 544 amino acids in length, and the predicted molecular weights (MWs) of deduced proteins ranged from 57.36 to 62.11 kDa. The isoelectric points (PIs) ranged from 6.77 to 9.32. Fifteen of the 24 UGTs had signal peptide (SP) sequences at the N-terminal end. To understand the evolutionary relationships of *C. suppressalis* UGT genes, a phylogenetic analysis was performed by combing these data with the UGT orthologs from the other four species. The 24 *C. suppressalis* UGTs were clustered into 11 families, of which four and nine UGTs were clustered within the UGT33 and UGT40 families, respectively. There were two UGTs in the UGT39 and UGT42 family, respectively, and only one each in the UGT34, UGT44, UGT45, UGT46, UGT47, UGT50, and UGT340 families (Figure 1).

**Figure 2:**
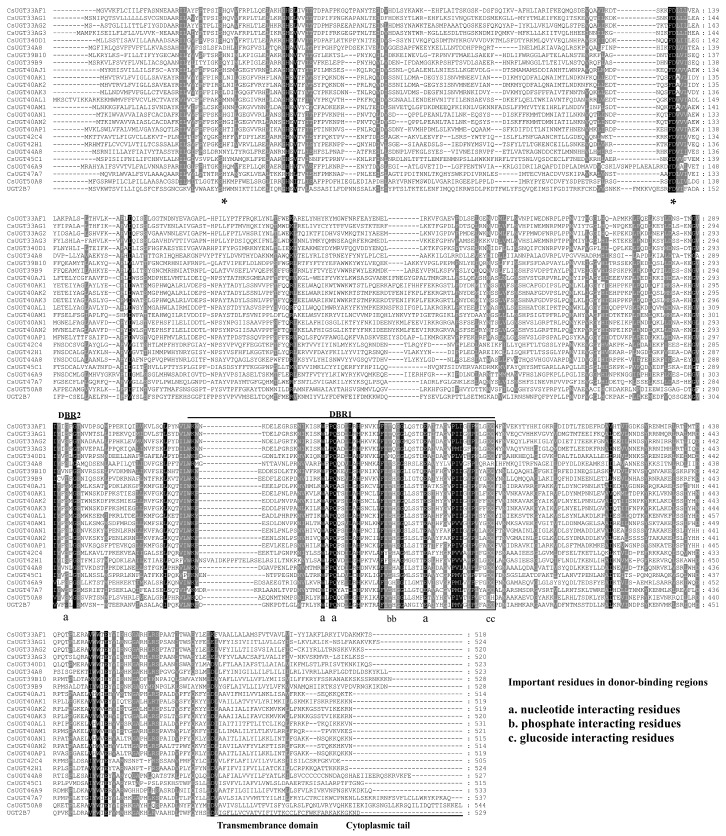
Multiple alignments of 24 UGTs in *C. suppressalis* with human *UGT2B7*. The transmembrane domain and cytoplasmic tails based on human *UGT2B7* are underlined. Asterisks (*) below alignments indicate the important catalytic residues (H and D). Black and grey background indicate that residues strictly conserved and residues well conserved, respectively. Two donor-binding regions (DBR1 and DBR2) are overlined. The signature motif is boxed. Several important residues interacting with the sugar donor are indicated by letters (a, b, and c) under the alignment.

The amino acid sequence alignments of the 24 UGTs with human *UGT2B7* revealed that these UGT proteins contained the two major parts: the highly variable N-terminal substrate-binding part, and the conserved C-terminal sugar donor-binding part. The two catalytic residues deduced from human *UGT2B7* modeling (H and D) were located in the N-terminal substrate-binding part. In addition, the two predicted sugar donor-binding regions (DBR1 and DBR2) were found in the middle of the C-terminal part, and three important residues in DBR interacting with the sugar donor were also conserved. The UGT signature motif sequence, (FVA)-(LIVMF)-(TS)-(HQ)-(SGAC)-G-X(2)-(STG)-X(2)-(DE)-X(6)-P-(LIVMFA)-(LIVMFA)-X(2)-P-(LMVFIQ)-X(2)-(DE)-Q (where X is any amino acid), was found in the middle of the C-terminal part [10]. The transmembrane domain, followed by cytoplasmic tails, was identified at the C-terminal end (Figure 2).

### 2.3. Screening of Overexpressed UGTs in C. suppressalis with Chlorantraniliprole Resistance

**Figure 3:**
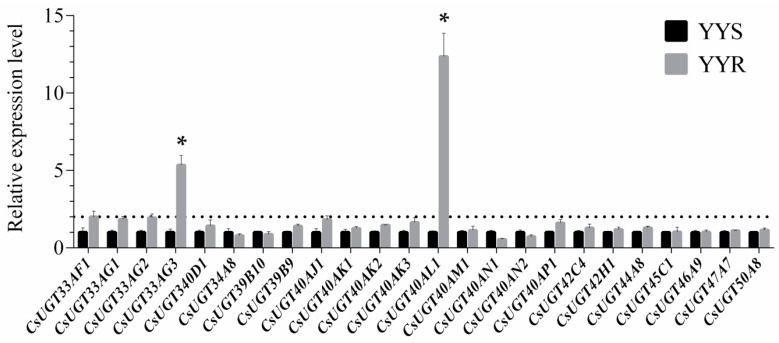
Relative expression levels of UGTs in YYS and YYR determined by real-time quantitative reverse transcription polymerase chain reaction (qRT-PCR). Data were normalized to the expression of *G3PDH* and *Actin A1*. Error bars represent the standard deviation (SD) from the mean of three independent replicates. Thirty fourth instar larvae were sampled for each biological replicate. Asterisks (*) indicate a significant difference in the expression levels of UGTs between YYS and YYR using Student’s *t*-test (*p* < 0.05).

Among the 24 UGT genes, *CsUGT40AL1* and *CsUGT33AG3*, which increased 12.36- and 5.34-fold in YYR, respectively, were significantly overexpressed in YYR compared to YYS (Figure 3). No significant differences between YYS and YYR were observed in the other 22 gene expressions.

### 2.4. Expression Patterns of Overexpressed UGTs in Different Tissues

**Figure 4:**
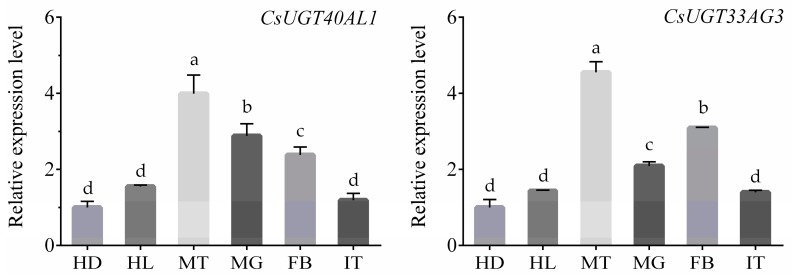
Expressions of *CsUGT40AL1* and *CsUGT33AG3* in different tissues of the fourth instar larvae of YYS. HD, head; HL, hemolymph; MT, Malpighian tubules; MG, midgut; FB, fat body; and IT, integument. The mRNA levels in the different tissues were normalized to the expressions in the head. Error bars represent the SD from the mean of three independent replicates. For each biological replicate, 30 fourth instar larvae were sampled. Different lowercase letters indicate significant differences among treatments as determined by one-way analysis of variance (ANOVA) followed by Tukey’s honest significant difference (Tukey’s HSD) test (*p* < 0.05).

To investigate the tissue-specific expression patterns of the two overexpressed UGTs, qRT-PCR was carried out using RNA isolated from the head, hemolymph, Malpighian tubules, midgut, fat body, and integument tissue samples of *C. suppressalis*. *CsUGT40AL1* and *CsUGT33AG3* showed high expression levels in the Malpighian tubules (3.99- and 4.55-fold, respectively), midgut (2.88- and 2.09-fold, respectively), and fat body (2.38- and 3.09-fold, respectively), followed by the hemolymph (1.55- and 1.44-fold, respectively) and integument (1.19- and 1.40-fold, respectively). However, the relative expressions of the two UGTs were lowest in the head (Figure 4). The expression patterns of *CsUGT40AL1* and *CsUGT33AG3* may reflect their specialized roles in insecticide resistance.

### 2.5. Confirmation of Overexpressed UGT Genes Function

**Figure 5:**
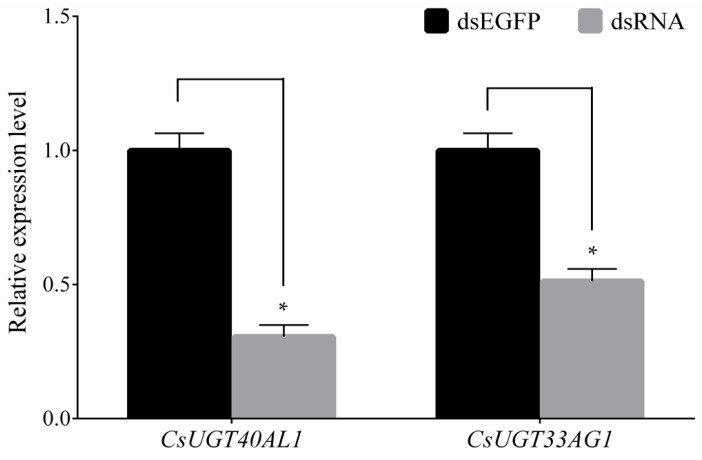
qRT-PCR analysis of UGT gene expression after individual UGT dsRNA injection in YYR of *C. suppressalis*. Data were normalized to the expression of *G3PDH* and *Actin A1*. Error bars represent the SD from the mean of three independent replicates. There were 30 fourth instar larvae for each biological replicate. Asterisks (*) indicate significant differences between treatments as determined by Student’s *t*-test (*p* < 0.05).

**Figure 6:**
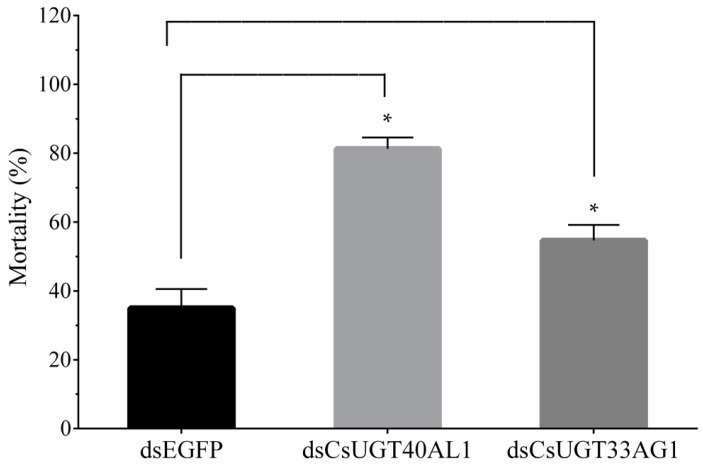
Mortality of chlorantraniliprole-treated YYR strain of *C. suppressalis* larvae after individual dsRNA injection. Error bars represent the SD from the mean of three independent replicates. For each biological replicate, 30 fourth instar larvae were sampled. Asterisks (*) indicate significant differences between treatments as determined by Student’s *t*-test (*p* < 0.05).

A further in vivo functional study was conducted using RNAi to silence the two overexpressed genes, followed by a chlorantraniliprole bioassay to evaluate the contribution of these genes to chlorantraniliprole resistance. After double-stranded RNA (dsRNA) injection, *CsUGT40AL1* and *CsUGT33AG3* expression greatly decreased by 69.34% and 48.74% compared to that after injection with double-stranded enhanced green fluorescent protein (dsEGFP), respectively (Figure 5). This indicated that dsRNA injection effectively silenced these target genes. The susceptibility of the dsRNA-injected YYR *C. suppressalis* to chlorantraniliprole was evaluated on the basis of fourth instar larval mortality. High mortality was observed after individual injection with *CsUGT40AL1* (81.33%) and *CsUGT33AG3* (54.67%), compared to that after dsEGFP injection (35.42%) (Figure 6). However, the mortalities showed no significant differences among the three dsRNA treatments in the absence of chlorantraniliprole (results not shown). These results suggested that the RNAi-mediated silencing of the two UGT genes could decrease the resistance of YYR to chlorantraniliprole.

## 3. Discussion

Since 2008, chlorantraniliprole has been registered and commercialized in China for agricultural usage to control the occurrence of several lepidopteran taxa, which provides a valid method for managing insecticide resistance [3]. The mode of its action can result in uncontrolled release of the calcium stores from muscle cells [7]. Although chlorantraniliprole is especially effective against *C. suppressalis*, frequent use of this insecticide has prompted rapid development of chlorantraniliprole resistance in this pest. A previous resistance monitoring study showed that *C. suppressalis* in the Jiangxi, Hunan, Henan, and Zhejiang provinces of China developed moderate to high resistance to chlorantraniliprole (64.10- to 249.60-fold) in the field [1,2,8]. In the present study, we found that *C. suppressalis* could develop high-level resistance to chlorantraniliprole in the laboratory under selection pressure, which was in line with previous findings [8,9]. The results indicated that *C. suppressalis* populations from different geographical areas seem to have a remarkable ability to develop resistance to this insecticide under continuous pressure. Additionally, several other lepidopteran pests, such as *P. xylostella*, *Spodoptera exigue*, and *Spodoptera litura*, developed high chlorantraniliprole resistance in China [18,19,20]. Despite the occurrence of resistance, chlorantraniliprole is still used in agriculture for lepidopteran pest management. To make good use of this insecticide and prolong its usage, an in-depth understanding of the resistance mechanisms is necessary. A susceptible YYS and a resistant YYR were established under intensive laboratory selection environments to decrease genetic diversity, which provided a genetic background to elucidate resistance mechanisms.

Understanding resistance mechanisms is a prerequisite for establishing resistance management strategies. An important metabolic resistance mechanism of chlorantraniliprole mediated by phase I enzyme P450s in *C. suppressalis* has been reported [9]; however, the increase in UGT enzyme activities is another main mechanism conferring insecticide resistance. The application of inhibitors is an effective way to verify an enzyme’s function. This study showed that the toxicities of chlorantraniliprole were significantly increased by the two UGT inhibitors (5-NU and SUL) in the YYR strains but not in the YYS strain, suggesting that enhanced UGTs enzyme activities may contribute to biochemical mechanisms of resistance in *C. suppressalis*. The two synergists had high potential for utilization in the management of chlorantraniliprole-resistant insects due to their significant synergistic effects. Therefore, a glycosylation by UGTs might play an important role in the detoxification of chlorantraniliprole in *C. suppressalis*.

Although UGT enzymes might be associated with chlorantraniliprole resistance in *C. suppressalis*, the specific genes that encoded this functional enzyme were not likely to have been previously identified. In this study, 24 UGT genes were identified by searching the *C. suppressalis* genome. The availability of whole genome and transcriptome sequences of various insects enables the characterization and comparative analysis of UGTs in these species. The total number of UGT genes identified was lower than that identified in three species, namely *Helicoverpa armigera* (40 genes), *Bombyx mori* (44 genes), and *Drosophila melanogaster* (34 genes), but higher than that in *Spodoptera littoralis* (11 genes) and similar to that in *P. xylostella* (23 genes) and *Athetis lepigone* (23 genes) [21,22,23,24,25,26]. Fifteen UGTs had signal peptide sequences at the N-terminal end. However, a signal peptide was absent from the other UGT genes, which were most likely non-secretory proteins, despite the complete sequence. Alignments of the *C. suppressalis* UGT amino acid sequences showed conserved domains, including DBR1 and DBR2 (important residues interacting with the sugar donor and catalytic residues), and the UGT motif signature sequences. These data suggested that *C. suppressalis* UGTs were likely active proteins, which is similar to observations reported for other lepidopteran taxa [25]. The evolutionary relationships of *C. suppressalis* UGTs suggest that the identified UGT transcripts form a complete UGT gene repertoire of *C. suppressalis*. The *C. suppressalis* UGTs were distributed into 11 families (i.e., UGT33, UGT34, UGT39, UGT40, UGT42, UGT44, UGT45, UGT46, UGT47, UGT50, and UGT340), which formed family clusters with other insect UGTs. Phylogenetic tree analysis of *C. suppressalis* UGTs revealed patterns of interspecific conservation and lineage-specific expansion of the gene families. Similar to other lepidopteran taxa, 4 and 9 UGTs were clustered in the UGT33 and UGT40 families, respectively, indicating that the divergence of these genes in the two families likely increased the range of compounds that could be detoxified or regulated by glycosylation [21,25]. Thus, these identified UGT genes in *C. suppressalis* could be used to further clarify their roles in chlorantraniliprole resistance.

The underlying UGTs that mediate metabolic resistance mechanisms were characterized by expression levels of UGT genes between the susceptible and resistant strains. In this study, *CsUGT40AL1* and *CsUGT33AG3* were significantly overexpressed in YYR, compared to YYS, which is in line with the synergism assays data, suggesting that overexpression of multiple UGT genes play a role in chlorantraniliprole resistance in *C. suppressalis*. The full-length cDNA sequences of the two UGT genes were obtained. The conserved signature motif position and conserved amino acid residues appeared to be diverse for each upregulated UGT gene, indicating the diversity of UGT genes involved in chlorantraniliprole resistance in *C. suppressalis*. The UGT33 and UGT40 family genes may have diverse functions, including detoxification of xenobiotics and insecticide resistance. Krempl et al. (2016) found that gossypol is partially metabolized by *UGT40D1* via glycosylation in *H. armigera* and *H. virescens*, which might be a crucial step in gossypol detoxification in generalist herbivores utilizing cotton as host plant [27]. Some UGTs from the two families were overexpressed in the adult antennae of *A. lepigone*, suggesting that they contribute to the degradation of sex pheromones and plant volatiles, and/or insecticide detoxification [26]. *UGT2B17* (renamed *UGT33AA4*) from the UGT33 family is involved in chlorantraniliprole resistance in *P. xylostella* [16,25]. The identified *CsUGT40AL1* and *CsUGT33AG3* sequences clustered into an own group separated from *UGT2B17* (renamed *UGT33AA4*) from *P. xylostella* in phylogenetic tree, suggesting the specificity of UGTs in different insect species. Our study, combined with these examples, confirmed contributions of *CsUGT40AL1* and *CsUGT33AG3* in chlorantraniliprole resistance in *C. suppressalis*. Expression of *CsUGT40AL1* and *CsUGT33AG3* was high in the Malpighian tubules, midgut, and fat body, followed by the hemolymph and the integument but was significantly lower in the head. The insect Malpighian tubules, fat body, and midgut are well-known to play crucial roles in the metabolism of xenobiotics and restrict the spread of the insecticide and minimize its toxic effects [28]. Similar expression patterns of UGT genes were reported for *P. xylostella* [16], *H. armigera* [21], and *B. mori* [22]. This suggests that the two UGT genes highly expressed in those tissues may be involved in sequestering chlorantraniliprole and in the detoxification processes.

To confirm a functional role of the overexpressed UGT genes in chlorantraniliprole resistance, we performed gene-silencing RNAi assays. Such technology has previously been used to analyze gene functions in *C. suppressalis* and has shown potential for developing new pest control measures [9,16,29]. Our results showed that dsRNA injection significantly silenced the target genes. This notable knockdown of the target genes resulted in a higher larval mortality rate than control in the resistant strain, indicating a loss of resistance. The RNAi results indicated both UGTs contributed to chlorantraniliprole resistance. But the expression of *CsUGT40AL1* was much higher than that of *CsUGT33AG3*, and tissue-specific expression patterns of *CsUGT40AL1* and *CsUGT33AG3* also showed significant differences. Therefore, further metabolism efficiencies were compared to evaluate the importance of the two UGT proteins in chlorantraniliprole resistance. The present study showed that UGTs mediated metabolic resistance of chlorantraniliprole in *C. suppressalis*, in addition to P450-mediated resistance.

## 4. Materials and Methods

### 4.1. Insects and Sample Preparation

The *C. suppressalis* colony was derived from a field population collected from a paddy field near Yuyao City, Zhejiang Province, China, in March 2016. After collection, a susceptible strain YYS was established by maintaining one part of the population on rice seedlings in the laboratory for 17 generations without exposure to insecticide. The rest of the population was continuously selected in the laboratory by treating fourth instar larvae with LC_50_ of chlorantraniliprole (using the diet surface overlay bioassay method [9]) for 17 generations to obtain a resistant strain of YYR. The *C. suppressalis* colony was reared on rice seedlings at 27 ± 1 °C with a humidity of 70–80% and 16-h light/8-h dark photoperiod in the laboratory.

Fourth instar larvae in YYS and YYR were collected to analyze tissue-specific expression patterns for the different strains. The fat body, midgut, hemolymph, integument, head, and Malpighian tubules were dissected from fourth instar larvae of the YYS strain, and washed with phosphate-buffered saline (PBS; 140 mM NaCl, 27 mM KCl, 8 mM Na_2_HPO_4_ and 1.5 mM KH_2_PO_4_ pH 7.4). Whole insect and tissue samples from those individuals were immediately frozen in liquid nitrogen and stored at −80 °C until RNA extraction. Each collection was replicated three times.

### 4.2. Toxicity and Synergism Bioassays

A stock solution of chlorantraniliprole (97.3%, TC; DuPont Trading Co., Ltd., Shanghai, China) was dissolved in *N*,*N*-dimethylformamide (Solarbio Science and Technology Co., Ltd., Beijing, China) and serially diluted into 5–6 concentrations with distilled water containing 0.1% Triton X-100 (Sinapharm Chemical Reagent Co., Ltd., Shanghai, China) as a cosolvent. The artificial diet was prepared as previously described [9]. The insecticide surface overlay diet was prepared by applying diluted chlorantraniliprole (100 μL) to the surface of the artificial diet (900 μL) in each well of a 24 well plate after the artificial diet solidified. A single fourth instar larva was transferred into each well when the liquid disappeared. Insects feeding the diet treated with water containing *N*,*N*-dimethylformamide and Triton X-100 were used as control. Each treatment was replicated three times for a total of 144 larvae per concentration. All treated insects were reared under the above rearing conditions. The mortality was checked after 72 h. Using a synergism bioassay, two UGT inhibitors, 5-NU and SUL (Sigma-Aldrich Trading Co., Ltd., Shanghai, China), were dissolved into a series of chlorantraniliprole concentrations with 0.1% Triton X-100 at a final concentration of 600 mg/L. The final concentrations of 5-NU and SUL were used in the maximum sublethal concentration that led to zero mortality in fourth instar larvae. Three replicates were performed for each concentration. All other procedures were the same as above. The LC_50_ values were calculated using probit analysis in the PoloPlus software (LeOra Software Inc., Petaluma, CA, USA). SRs were calculated as the LC_50_ values of the insecticide alone divided by the LC_50_ values of the insecticide with the synergist treatments. A significant difference was deemed to exist between LC_50_s when their 95% CL did not overlap.

### 4.3. RNA Isolation and cDNA Synthesis

Total RNA was extracted from samples using Trizol reagent (Life Technologies, Carlsbad, CA, USA) following the manufacturer’s instructions. The quality and quantity of RNA were checked using agarose gel electrophoresis (1.3%) and Nanodrop spectrophotometry (Thermo Fisher Scientific, Waltham, MA, USA). First-strand cDNA was synthesized by HiScript II Q RT SuperMix for qPCR (+gDNA wiper) (Vazyme biotech Co., Ltd., Nanjing, China) according to the manufacturer’s protocol.

### 4.4. Identification of UGT Genes and Phylogenetic Analysis

UGT gene sequences were obtained by querying genome databases of *C. suppressalis* (http://www.insect-genome.com/) with annotated gene names [30]. Cap3 software was used to identify the overlaps between different sequences and to remove redundant fragments (http://doua.prabi.fr/software/cap3). Uridine diphosphate glycosyltransferase conserved domains ‘UDPGT’ of UGT genes were analyzed by using the SMART online software (http://smart.embl-heidelberg.de/) [31]. Each of the putative UGT sequences was used as a query to search the NCBI non-redundant protein database using BLASTX to validate further the potential UGTs (http://www.ncbi.nlm.nih.gov/). Finally, the putative 24 UGT genes with complete ORFs were obtained. The accuracy of these putative UGT genes was substantiated by RT-PCR using the primers detailed in Table S1 (Supplementary Materials), and these were submitted to GenBank. The complete ORFs of *C. suppressalis* UGT genes were predicted using the ORF finder tool (http://www.ncbi.nlm.nih.gov/gorf/gorf.html). The SPs were predicted using SignalP 4.1 (http://www.cbs.dtu.dk/services/SignalP/). The PIs and MWs of deduced proteins were calculated using the ExPASy Compute pI/Mw tool (http://web.expasy.org/compute_pi/). A set of 163 UGT gene sequences from 5 species, namely *S. exigua* (32 sequences), *B. mori* (44 sequences), *H. armigera* (40 sequences), *P. xylostella* (23 sequences) and *C. suppressalis* (24 sequences), were obtained from the NCBI database. The names and accession numbers of the genes used for phylogenetic tree building are listed in Table S2 (Supplementary Materials). Amino acid sequences were aligned with ClustalX 2.0 and unrooted trees were constructed by MEGA 5.0 using the neighbor-joining method with Poisson correction of distances [32,33,34]. Node support was assessed using a bootstrap procedure based on 1000 replicates.

### 4.5. Expression Patterns of UGT Genes in Resistant and Susceptible Strains

qRT-PCR was performed to determine the expression patterns of UGT genes in different strains and tissues using an Applied Biosystems™ QuantStudio™ 6 Flex Real-Time PCR System. Primers were designed by Beacon Designer 7.0 (Premier Biosoft International, Palo Alto, CA, USA) and are listed in Table S3 (Supplementary Materials). A ChamQ^TM^ SYBR^®^ qPCR Master Mix (Vazyme biotech Co., Ltd., Nanjing, China) was used according to the manufacturer’s protocol. Normalization of each experimental gene expression level was conducted based on the geometric mean of two selected reference genes, *G3PDH* and *Actin A1* [8]. The expression stability of the two reference genes was analyzed with geNorm Software [35]. Data were calculated according to the 2^−ΔΔ*C*t^ method.

### 4.6. Functional Analysis of Overexpressed UGT Genes with RNAi

Total RNA was extracted using Trizol reagent (Invitrogen, Carlsbad, CA, USA), and the first-strand cDNA was synthesized as previously described. The specific primers for dsRNA synthesis were designed on the basis of sequences of overexpressed UGT genes and the fragment sequence of an enhanced green fluorescent protein (EGFP), which contained T7 polymerase promoter sequence at both ends (Table S4, Supplementary Materials). The PCR products amplified by RT-PCR were purified using Wizard^®^SV gel and the PCR clean-up system (Promega, Madison, WI, USA) according to the manufacturer’s protocol and were used as templates for in vitro transcription using the T7 RiboMAX^TM^ Express RNAi System (Promega, Madison, WI, USA) to synthesize dsRNA. All dsRNAs were resuspended in nuclease-free water and quantified spectrophotometrically. These dsRNAs were examined by agarose gel electrophoresis to verify that the products exhibited the expected sizes in a single band. Then they were stored at −80 °C until use.

RNAi experiments were performed using the dsRNA microinjection method. The dsRNA was dissolved in diethyl pyrocarbonate-treated water to a final concentration of 4 µg/µL. Then dsRNA (1 µL, 4 µg) was injected into the larvae using a microneedle. The needles were pulled from glass capillaries (outer diameter, 1 mm; inner diameter, 0.5 mm) using a micropipette puller (Model P-97, Sutter Instruments Co., Novato, CA, USA). The needles were held still at the injection point for 30 s to avoid small interfering RNA leakage. The dsRNA injection was performed using an Eppendorf InjectMan NI 2 microinjection system (Eppendorf, Hamburg, Germany). Thirty fourth instar larvae in YYR were used for each treatment, and all experiments were performed in triplicate.

Three hours after interfering, 30 surviving larvae from each treatment were treated according to the preliminary bioassay method described above. The treated *C. suppressalis* were kept under the rearing conditions described above, and the mortality was checked after 72 h.

To estimate the target gene silencing effect following RNAi, qRT-PCR was used to measure the expression levels of the target genes in YYR. qRT-PCR was performed as previously described. The primers used are listed in Table S4 (Supplementary Materials).

### 4.7. Statistical Analysis

Data are presented as mean ± standard deviation (SD). Student’s *t*-test was used for two sample comparisons. One-way analysis of variance (ANOVA) followed by Tukey’s honest significant difference (Tukey’s HSD) test was used for multiple group comparisons. All statistical analyses were performed using SPSS 20.0 software (SPSS Inc., Chicago, IL, USA). *p* < 0.05 was considered statistically significant.

## 5. Conclusions

The present study strongly supports the conclusion that the overexpression of UGTs is responsible for resistance to chlorantraniliprole in *C. suppressalis*. These results, in conjunction with the involvement of UGT in chlorantraniliprole resistance of *P. xylostella*, provide evidence that overexpression of UGTs in chlorantraniliprole resistance may be a common mechanism in lepidopteran pests. Future pest management decisions using chlorantraniliprole to control lepidopterans should take this into account.

## Figures and Tables

**Table 1:** Synergism effects of 5-NU and SUL on the toxicities of chlorantraniliprole in the YYS and YYR strains.

Strain	Treatment	LC_50_ (95% CL) (mg·L^−1^)	Slope ± SE	*x* ^2^	RR	SR
YYS	Chlorantraniliprole	12.76 (11.08–14.76)	2.72 ± 0.31	5.96	1.00	1.00
	Chlorantraniliprole + 5-NU	10.48 (8.95–12.27)	2.42 ± 0.35	5.17	-	1.22
	Chlorantraniliprole + SUL	11.27 (9.76–13.03)	2.70 ± 0.32	7.72	-	1.13
YYR	Chlorantraniliprole	565.48 (510.42–626.47)	3.67 ± 0.22	7.31	44.32	1.00
	Chlorantraniliprole + 5-NU	167.47 (146.78–191.08)	2.97 ± 0.29	2.68	-	3.38 *
	Chlorantraniliprole + SUL	158.71 (139.32–180.79)	3.08 ± 0.28	7.75	-	3.56 *

* Indicates statistical significance. CL, Confidence limits; RR = LC_50_ of YYR strain/LC_50_ of YYS strain; SR = LC_50_ of chlorantraniliprole/LC_50_ of chlorantraniliprole + synergist.

**Table 2:** Characteristics of the UGT genes identified in *C. suppressalis*.

Gene Name	Accession Number	ORF Size (aa)	PI/MW (KDa)	SP	‘UDPGT’ Domain	Best Blastx Match		
						Species Name ^1^	GenBank Accession Number	Identity
*CsUGT33AF1*	MK135471	518	7.77/59.32	1–19	22–517	Ha	XP_021197637.1	62%
*CsUGT33AG1*	MK135472	524	6.91/60.06	1–22	24–517	Pp	XP_013139422.1	61%
*CsUGT33AG2*	MK135473	520	6.77/59.27	1–20	24–517	Ha	AEW43120.1	55%
*CsUGT33AG3*	MK135474	520	9.14/59.67	1–23	25–519	Ha	AEW43118.1	57%
*CsUGT340D1*	MK135475	523	7.24/59.54	1–19	21–519	Bm	NP_001243963.1	52%
*CsUGT34A8*	MK135476	523	8.30/59.31	–	22–510	At	XP_013184861.1	62%
*CsUGT39B10*	MK135477	528	9.00/61.43	–	110–517	Bm	NP_001243980.1	69%
*CsUGT39B9*	MK135478	528	9.01/60.69	1–21	95–516	Se	ANI21999.1	66%
*CsUGT40AJ1*	MK135479	514	8.87/58.20	–	19–513	Ha	XP_021183292.1	56%
*CsUGT40AK1*	MK135480	519	8.39/59.76	–	29–518	Ha	AEW43128.1	52%
*CsUGT40AK2*	MK135481	519	8.83/59.17	–	37–515	Ha	AEW43128.1	52%
*CsUGT40AK3*	MK135482	520	8.76/59.33	1–18	27–516	Ha	XP_021191114.1	54%
*CsUGT40AL1*	MK135483	531	9.32/61.01	–	32–531	Ha	AEW43128.1	54%
*CsUGT40AM1*	MK135484	521	8.13/59.30	1–20	22–520	Dp	OWR51610.1	59%
*CsUGT40AN1*	MK135485	515	9.00/57.36	–	22–514	At	XP_013186135.1	56%
*CsUGT40AN2*	MK135486	514	8.94/57.42	–	22–513	At	XP_013189977.1	56%
*CsUGT40AP1*	MK135487	519	9.22/58.68	1–19	24–518	Bm	XP_012550238.1	56%
*CsUGT42C4*	MK135488	505	7.63/57.49	1–21	24–500	Se	ANI22012.1	65%
*CsUGT42H1*	MK135489	524	8.67/59.39	1–22	24–523	Pm	XP_014358643.1	61%
*CsUGT44A8*	MK135490	515	6.79/59.79	–	30–511	Ha	XP_021186279.1	62%
*CsUGT45C1*	MK135491	515	8.33/58.49	1–19	24–502	Pp	XP_013141171.1	54%
*CsUGT46A9*	MK135492	533	8.96/60.29	1–23	27–528	At	XP_013183121.1	66%
*CsUGT47A7*	MK135493	530	8.94/60.44	1–18	19–510	At	XP_013192144.1	78%
*CsUGT50A8*	MK135494	544	8.88/62.11	1–21	79–516	Ha	XP_021184423.1	74%

ORF, open reading frame; MW, molecular weight; PI, isoelectric point; SP, signal peptide. ^1^ Se (*Spodoptera exigua*), Ha (*Helicoverpa armigera*), Pp (*Papilio polytes*), Bm (*Bombyx mori*), At (*Amyelois transitella*), Dp (*Danaus plexippus*), Pm (*Papilio machaon*).

## Supplementary Materials

Supplementary materials can be found at https://www.mdpi.com/1422-0067/20/5/1064/s1.

## Abbreviations

UGT	Uridine diphosphate glycosyltransferase
UDP	Uridine diphosphate
RyR	Ryanodine receptor
RNAi	RNA interference
dsRNA	Double-stranded RNA
5-NU	5-nitrouracil
SUL	Sulfinpyrazone
RR	Resistance ratio
SR	Synergism ratio
CL	Confidence limits
ORF	Open reading frame
MW	Molecular weight
PI	Isoelectric point
SP	Signal peptide
EGFP	Enhanced green fluorescent protein
qRT-PCR	Real-time quantitative reverse transcription polymerase chain reaction
RT-PCR	Reverse transcription polymerase chain reaction
PBS	Phosphate-buffered saline
SD	Standard deviation
ANOVA	Analysis of variance
Tukey’s HSD	Tukey’s honest significant difference

## References

[1] Lu Y.H., Wang G.R., Zhong L.Q., Zhang F.C., Bai Q., Zheng X.S., Lu Z.X. (2017). Resistance monitoring of *Chilo suppressalis* (Walker) (Lepidoptera: Crambidae) to chlorantraniliprole in eight field populations from east and central China. Crop. Prot..

[2] Huang S.J., Chen Q., Qin W.J., Sun Y., Qin H.G. (2017). Resistance monitoring of four insecticides and a description of an artificial diet incorporation method for *Chilo suppressalis* (Lepidoptera: Crambidae). J. Econ. Entomol..

[3] Su J.Y., Zhang Z.Z., Wu M., Gao C.F. (2014). Geographic susceptibility of *Chilo suppressalis* Walker (Lepidoptera: Crambidae), to chlorantraniliprole in China. Pest Manag. Sci..

[4] Sun Y., Huang S.J., Wang S.P., Guo D.H., Ge C., Xiao H.M., Jie W.C., Yang Q.P., Teng X.L., Li F. (2017). Large-scale identification of differentially expressed genes during pupa development reveals solute carrier gene is essential for pupal pigmentation in *Chilo suppressalis*. J. Insect Physiol..

[5] Cordova D., Benner E.A., Sacher M.D., Rauh J.J., Sopa J.S., Lahm G.P., Selby T.P., Stevenson T.M., Flexner L., Gutteridge S. (2006). Anthranilic diamides: A new class of insecticides with a novel mode of action, ryanodine receptor activation. Pestic. Biochem. Physiol..

[6] He Y.P., Zhang J.F., Gao C.F., Su J.Y., Chen J.M., Shen J.L. (2013). Regression analysis of dynamics of insecticide resistance in field populations of *Chilo suppressalis* (Lepidoptera: Crambidae) during 2002–2011 in China. J. Econ. Entomol..

[7] Yao R., Zhao D.D., Zhang S., Zhou L.Q., Wang X., Gao C.F., Wu S.F. (2017). Monitoring and mechanisms of insecticide resistance in *Chilo suppressalis* (Lepidoptera: Crambidae), with special reference to diamides. Pest Manag. Sci..

[8] Sun Y., Xu L., Chen Q., Qin W.J., Huang S.J., Jiang Y., Qin H.G. (2018). Chlorantraniliprole resistance and its biochemical and new molecular target mechanisms in laboratory and field strains of *Chilo suppressalis* (Walker). Pest Manag. Sci..

[9] Xu L., Zhao J., Sun Y., Xu D., Xu G., Xu X., Zhang Y., Huang S., Han Z., Gu Z. (2019). Constitutive overexpression of cytochrome P450 monooxygenase genes contributes to chlorantraniliprole resistance in *Chilo suppressalis* (Walker). Pest Manag. Sci..

[10] Mackenzie P.I., Owens I.S., Burchell B., Bock K.W., Bélanger A., Belanger A., Fournel-Gigleux S., Green M., Hum D.W., Iyanagi T. (1997). The UDP glycosyltransferase gene superfamily: Recommended nomenclature update based on evolutionary divergence. Pharmacogenetics.

[11] Bock K.W. (2003). Vertebrate UDP-glucuronosyltransferases: functional and evolutionary aspects. Biochem. Pharmacol..

[12] Bull D.L., Whitten C.J. (1972). Factors influencing organophosphorus insecticide resistance in tobacco budworms. J. Agric. Food. Chem..

[13] Lee S.W., Ohta K., Tashiro S., Shono T. (2006). Metabolic resistance mechanisms of the housefly (*Musca domestica*) resistant to pyraclofos. Pestic. Biochem. Physiol..

[14] Kaplanoglu E., Chapman P., Scott I.M., Donly C. (2017). Overexpression of a cytochrome P450 and a UDP-glycosyltransferase is associated with imidacloprid resistance in the Colorado potato beetle, *Leptinotarsa decemlineata*. Sci. Rep..

[15] Pan Y.O., Tian F.Y., Wei X., Wu Y.Q., Gao X.W., Xi J.H., Shang Q.L. (2018). Thiamethoxam resistance in *Aphis gossypii* glover relies on multiple UDP-glucuronosyltransferases. Front. Physiol..

[16] Li X.X., Zhu B., Gao X.W., Liang P. (2017). Over-expression of UDP-glycosyltransferase gene *UGT2B17* is involved in chlorantraniliprole resistance in *Plutella xylostella* (L.). Pest Manag. Sci..

[17] Mackenzie P.I., Bock K.W., Burchell B., Guillemette C., Ikushiro S., Iyanagi T., Miners J.O., Owens I.S., Nebert D.W. (2005). Nomenclature update for the mammalian UDP glycosyltransferase (*UGT*) gene superfamily. Pharmacogenet. Genom..

[18] Wang X.L., Wu Y.D. (2012). High levels of resistance to chlorantraniliprole evolved in field populations of *Plutella xylostella*. J. Econ. Entomol..

[19] Lai T.C., Li J., Su J.Y. (2011). Monitoring of beet armyworm *Spodoptera exigua* (Lepidoptera: Noctuidae) resistance to chlorantraniliprole in China. Pestic. Biochem. Physiol..

[20] Su J.Y., Lai T.C., Li J. (2012). Susceptibility of field populations of *Spodoptera litura* (Fabricius) (Lepidoptera: Noctuidae) in China to chlorantraniliprole and the activities of detoxification enzymes. Crop Prot..

[21] Ahn S.J., Vogel H., Heckel D.G. (2012). Comparative analysis of the UDP-glycosyltransferase multigene family in insects. Insect Biochem. Mol. Biol..

[22] Huang F.F., Chai C.L., Zhang Z., Liu Z.H., Dai F.Y., Lu C., Xiang Z.H. (2008). The UDP-glucosyltransferase multigene family in *Bombyx mori*. BMC Genomics.

[23] Luque T., O’Reilly D.R. (2002). Functional and phylogenetic analyses of a putative *Drosophila melanogaster* UDP-glycosyltransferase gene. Insect Biochem. Mol. Biol..

[24] Bozzolan F., Siaussat D., Maria A., Durand N., Pottier M.A., Chertemps T., Maïbèche-Coisne M. (2014). Antennal uridine diphosphate (UDP)-glycosyltransferases in a pest insect: diversity and putative function in odorant and xenobiotics clearance. Insect Mol. Biol..

[25] Li X., Shi H., Gao X., Liang P. (2018). Characterization of UDP-glucuronosyltransferase genes and their possible roles in multi-insecticide resistance in *Plutella xylostella* (L.). Pest Manag. Sci..

[26] Zhang Y.N., Ma J.F., Xu L., Dong Z.P., Xu J.W., Li M.Y., Zhu X.Y. (2017). Identification and expression patterns of UDP-glycosyltransferase (UGT) genes from insect pest *Athetis lepigone* (Lepidoptera: Noctuidae). J. Asia-Pac. Entomol..

[27] Krempl C., Sporer T., Reichelt M., Ahn S., Heidel-Fischer H., Vogel H., Heckel D.G., Joußen N. (2016). Potential detoxification of gossypol by UDP-glycosyltransferases in the two Heliothine moth species *Helicoverpa armigera* and *Heliothis virescens*. Insect Biochem. Mol. Biol..

[28] Xu L., Zhao C.Q., Xu D.J., Xu G.C., Xu X.L., Han Z.J., Zhang Y.N., Gu Z.Y. (2017). RNAi suppression of nuclear receptor genes results in increased susceptibility to sulfoxaflor in brown planthopper, *Nilaparvata lugens*. J. Asia-Pac. Entomol..

[29] Tian F., Wang Z., Li C., Liu J., Zeng X. (2019). UDP-Glycosyltransferases are involved in imidacloprid resistance in the Asian citrus psyllid, *Diaphorina citri* (Hemiptera: Lividae). Pestic. Biochem. Physiol..

[30] Yin C.L., Shen G.Y., Guo D.H., Wang S.P., Ma X.Z., Xiao H.M., Liu J.D., Zhang Z., Liu Y., Zhang Y.Q. (2016). InsectBase: a resource for insect genomes and transcriptomes. Nucleic Acids Res..

[31] Wang S., Liu Y., Zhou J.J., Yi J.K., Pan Y., Wang J., Zhang X.X., Wang J.X., Yang S., Xi J.H. (2018). Identification and tissue expression profiling of candidate UDP-glycosyltransferase genes expressed in *Holotrichia parallela* motschulsky antennae. Bull. Entomol. Res..

[32] Larkin M.A., Blackshields G., Brown N.P., Chenna R., McGettigan P.A., McWilliam H., Valentin F., Wallace I.M., Wilm A., Lopez R. (2007). Clustal W and Clustal X version 2.0. Bioinformatics.

[33] Tamura K., Peterson D., Peterson N., Stecher G., Nei M., Kumar S. (2011). MEGA5: molecular gvolutionary genetics analysis using maximum likelihood, evolutionary distance, and maximum parsimony methods. Mol. Biol. Evol..

[34] Thompson J.D., Higgins D.G., Gibson T.J. (1994). Clustal W: improving the sensitivity of progressive multiple sequence alignment through sequence weighting, position-specific gap penalties and weight matrix choice. Nucleic Acids Res..

[35] Hellemans J., Mortier G., Paepe D.A., Speleman F., Vandesompele J. (2007). qBase relative quantification framework and software for management and automated analysis of real-time quantitative PCR data. Genome Biol..

