# Supplementary material for: UDP-Glycosyltransferase Genes in the Striped Rice Stem Borer, Chilo suppressalis (Walker), and Their Contribution to Chlorantraniliprole Resistance
Source: Int J Mol Sci. 2019 Mar 1;20(5):1064. doi: 10.3390/ijms20051064 (PMC6429375; doi:10.3390/ijms20051064)
Supplement: Supplementary file 1 [file ijms-20-01064-s001.pdf]

Table S1. Primers used in RT-PCR for *C. suppressalis* UGT genes.

Gene name	Forward primer sequence (5'-3')	Reverse primer sequence (5'-3')
CsUGT33AF1	ATGGGTGTAGTGAAGTTTTTGT	TTAGCTAGTTTTCATTTTAGCGT
CsUGT33AG1	ATGTCTAATATTCCGCAAACTTC	TCAGCTAAATTTAACCTTAGCCT
CsUGT33AG2	ATGTCTGCTTCAACCGGTCT	TTAACTCTTTTTAACCTTTTTACT
CsUGT33AG3	ATGGCGATGCCAAAAATTTCCG	CTAACTAGATTTCAACTTTATACTT
CsUGT340D1	ATGTTACTATCGAGCACAATT	TCAACTTTGTTTTATTTTATTTTTTG
CsUGT34A8	ATGGGTTTCATCAGATTGCAAA	TCATAACAATTTGTATTTGCTGT
CsUGT39B10	ATGTTAAAGAGATACTTGAAGTC	TCAGTTTTTCTTTTTATTTCTTGA
CsUGT39B9	ATGTCTCGAAAAGTGTTGTTTA	TTAATTGTCCTTTATTTTACCATT
CsUGT40AJ1	ATGTATAAACATATTAGTGTAATA	TTATTTCGTCTTTTGTTTCTTCT
CsUGT40AK1	ATGCACGTGACCCGTGTTTT	TCGAAGAGACCAAAAGAAAAGA
CsUGT40AK2	ATGCATGTGACCCGTGTTTT	TCAATTCCTCTTTTCTTTTATTAC
CsUGT40AK3	ATGCTTTTGAACGATGTTATGT	TCAATTCCTCTTTTCTTTTATTAC
CsUGT40AL1	ATGAAATCGTGCACAGTCATTAA	TTAATTCACCTTCTTCTTTATTTGT
CsUGT40AM1	ATGCTGAATAAGAAAGGGTTC	TTAACTCTCTTTAACTTTCACAG
CsUGT40AN1	ATGACAAAAATATGGGTTGCTG	TTAGTTTTTCTTTTGTTTCTTCTG
CsUGT40AN2	ATGACAAAAATATGGGTTGCTG	TTAGTTGACCTTATGTTTCTTTT
CsUGT40AP1	ATGGTGAAGTTGTCTTGGCTTG	TTAGTTTTTCTTTTGTTTCTTCTGA
CsUGT42C4	ATGAAAACCACGGTTGCCGTT	TCATCGACTTTACCTGGTCG
CsUGT42H1	ATGCGTCATATGACATTTTTATG	CTAACTTCTCTTTTTCTTAGTCG
CsUGT44A8	ATGTCCCGCAACATAATACTC	TTATTCGAACTTGACTCGTTTA
CsUGT45C1	ATGAGTCCGATATCAATAATTTT	CTATCCATTACCGGTAAAGG
CsUGT46A9	ATGCGAGCGCATTACGCGA	TCACTGCAACTTATCTTTAACTT
CsUGT47A7	ATGCAGGTGCGGCTATGG	TCATTGAGCTTTTGGTTTATAC
CsUGT50A8	ATGTTCGGGTCAAGGTGGC	TTACAGCTCCTTCTTTGATATC

Table S2. Accession numbers for amino acid sequences of UGTs used in phylogenetic analysis.

Species	Protein name	Accession number	Species	Protein name	Accession number	Species	Protein name	Accession number
	SeUGT33B14	ANI21986		BmUGT39B1	AEW43161		HaUGT40D2	AEW43124
	SeUGT33B15	ANI21987		BmUGT39C1	AEW43162		HaUGT40F1	AEW43125
	SeUGT33B16	ANI21988		BmUGT40A1	AEW43163		HaUGT40F2	AEW43126
	SeUGT33F5	ANI21989		BmUGT40B1	AEW43164		HaUGT40L1	AEW43127
	SeUGT33F6	ANI21990		BmUGT40B2P	AEW43165		HaUGT40M1	AEW43128
	SeUGT33F7	ANI21991		BmUGT40B3	AEW43166		HaUGT40Q1	AEW43129
	SeUGT33F8	ANI21992		BmUGT40B4	AEW43167		HaUGT40R1	AEW43130
	SeUGT33J3	ANI21993		BmUGT40G1	AEW43168		HaUGT41B1	AEW43131
	SeUGT33T3	ANI21994		BmUGT40G2	AEW43169		HaUGT41B2	AEW43132
	SeUGT33V1	ANI21995		BmUGT40H1	AEW43170	*Helicoverpa armigera*	HaUGT41B3	AEW43133
	SeUGT33V2	ANI21996		BmUGT40K1	AEW43171		HaUGT41D1	AEW43134
	SeUGT33V3	ANI21997		BmUGT40N1	AEW43172		HaUGT42B2	AEW43135
Spodoptera exigua	SeUGT33V4	ANI21998	*Bombyx mori*	BmUGT40P1	AEW43173		HaUGT42C1	AEW43136
	SeUGT39B4	ANI21999		BmUGT40S1	AEW43174		HaUGT43A1	AEW43137
	SeUGT40D4	ANI22000		BmUGT41A1	AEW43175		HaUGT44A2	AEW43138
	SeUGT40D5	ANI22001		BmUGT41A2	AEW43176		HaUGT46A3	AEW43139
	SeUGT40F3	ANI22002		BmUGT41A3	AEW43177		HaUGT46A4	AEW43140
	SeUGT40F4	ANI22003		BmUGT42A1	AEW43178		HaUGT46B1	AEW43141
	SeUGT40F5	ANI22004		BmUGT42A2	AEW43179		HaUGT47A2	AEW43142
	SeUGT40L3	ANI22005		BmUGT42B1	AEW43180		HaUGT48A1	AEW43143
	SeUGT40M2	ANI22006		BmUGT43B1	AEW43181		HaUGT50A2	AEW43144
	SeUGT40M3	ANI22007		BmUGT44A1	AEW43182		PxUGT33Y1-1	AUC64268
	SeUGT40Q2	ANI22008		BmUGT46A1	AEW43183	*Plutella xylostella*	PxUGT40Y1	AUC64269
	SeUGT40R4	ANI22009		BmUGT46A2	AEW43184		PxUGT47A5	AUC64270
	SeUGT40U2	ANI22010		BmUGT46C2	AEW43185		PxUGT41F1	AUC64271
	SeUGT42B5	ANI22011		BmUGT47A1	AEW43186		PxUGT46E1	AUC64272

	SeUGT42C2	ANI22012		BmUGT48C1	AEW43187		PxUGT42E1	AUC64273
	SeUGT43A2	ANI22013		HaUGT33B1	AEW43105		PxUGT42E2	AUC64274
	SeUGT44A5	ANI22014		HaUGT33B2	AEW43106		PxUGT39B8	AUC64275
	SeUGT46A7	ANI22015		HaUGT33B3	AEW43107		PxUGT40W1	AUC64276
	SeUGT48D1	ANI22016		HaUGT33B4	AEW43108		PxUGT33AA4	AUC64277
	SeUGT50A5	ANI22017		HaUGT33B5	AEW43109		PxUGT42F1	AUC64278
	BmUGT33D1	AEW43145		HaUGT33B7	AEW43110		PxUGT39A2	AUC64279
	BmUGT33D2	AEW43146		HaUGT33B8	AEW43111		PxUGT44A6	AUC64280
	BmUGT33D3	AEW43147		HaUGT33B9	AEW43112		PxUGT40V1	AUC64281
	BmUGT33D4	AEW43148		HaUGT33B11	AEW43113		PxUGT33AB1-1	AUC64282
	BmUGT33D5	AEW43149	***Helicoverpa armigera***	HaUGT33B12	AEW43114		PxUGT33Y1-2	AUC64283
	BmUGT33D6	AEW43150		HaUGT33F1	AEW43115		PxUGT34A5	AUC64284
Bombyx mori	BmUGT33D7	AEW43151		HaUGT33F2	AEW43116		PxUGT33AB1-2	AUC64285
	BmUGT33D8	AEW43152		HaUGT33F3	AEW43117		PxUGT45B1	AUC64286
	BmUGT33k1	AEW43153		HaUGT33J1	AEW43118		PxUGT33W1	AUC64287
	BmUGT33N1	AEW43154		HaUGT33M1	AEW43119		PxUGT50A6	AUC64288
	BmUGT33Q1	AEW43155		HaUGT33T1	AEW43120		PxUGT33AA1	AUC64289
	BmUGT33R1	AEW43156		HaUGT34A3	AEW43121		PxUGT40X1	AUC64290
	BmUGT33R2	AEW43157		HaUGT39B2	AEW43122			
	BmUGT34A2	AEW43160		HaUGT40D1	AEW43123			

Table S3. Primers used in qRT-PCR for *C. suppressalis* UGT genes.

	Gene name	Forward primer sequence (5'-3')	Reverse primer sequence (5'-3')
UGT genes	*CsUGT33AF1*	CATGGGCTGGTTCAATCGTT	GGCAAGGGACGATTGTCTTC
	CsUGT33AG1	CCACTCCTTCTATCAGCCATCA	GCGCTTTTCCATTCGGG
	CsUGT33AG2	ATGCCGCGCGAATATTG	CTGGTGCTTCTTCTCTTGAAAAC
	CsUGT33AG3	GTGTTCTCCAAACTCCCATACG	GGCCCGGCAATTCATCT
	CsUGT340D1	CCACCCTACAACATCACCGA	TACAGCGGGATGGTTCAGTT
	CsUGT34A8	TGAACTTGCCGCGTAATGTGG	CCTCGCTCTTTGCAGTGGGA
	CsUGT39B10	TCTCACGGAGGATTGATCGG	TTCGCAGGCTCTGTTCATTG
	CsUGT39B9	AGCATCGGACCACAATTTCG	GACCGTTGCCAACTGAAGAG
	CsUGT40AJ1	GGTCGCCTCTTGCCAAGTTT	TGGCAGCGGCTTAACTTCCT
	CsUGT40AK1	AGAAGCCGTTTACTGGGTGGA	AAATGGCGACCAGGTCCAGA
	CsUGT40AK2	ATGTTCCAGCCAACGTGAAG	AAGACAGTTGACCTCCGTGT
	CsUGT40AK3	TGGGTCCTCATTGGCAAGCA	GGACAGATAGTCCGCCGTGT
	CsUGT40AL1	GCCGACCTTCTGGAAACGGA	TGCGGGATTTGTGGGATCGT
	CsUGT40AM1	ACTGTCGACGACGGAAGCAA	GCTGAAAGTTCACCAGCCATGT
	CsUGT40AN1	CATTGGTCCTGGGCAACTCC	TTCACGTTCGAACCCAGGCT
	CsUGT40AN2	AGCCCTAACCACACCCTGG	TTTGCGTAGATTTTCTCCCG
	CsUGT40AP1	TGGTGCTGAGGCTGATCGAC	AGCGGCGCAAGATCAGAGTA
	CsUGT42C4	ACCTGTGGTAGCAATGCCGAT	GACGATCGTGCCACACTTGG
	CsUGT42H1	ATGCCCATTCTTGGAGACCA	CGTGTCCAAAGGTGACAGAG
	CsUGT44A8	AAGACTCCACTCTGCCGAAA	ACCGGCATGGGATATGAACA
	CsUGT45C1	CCAGTGCCACAGGATGTAGA	GCGGTGACATTAGACTGCTG
	CsUGT46A9	GTTATCGAGCGGACTGATGC	TCCAGGAACGTCATCTTGCT
	CsUGT47A7	CTCAACGAACAACTGGGCAA	CCGATCACAGAAAGCGTGTT
	CsUGT50A8	TTACCACCGCTGGACATCAT	CAACACCCATAGCGCATTCA
Reference genes	*G3PDH*	GTTGTGCCTCACCAATTTGTCAG	GCCACCTTCAGCGATGTCG
	Actin A1	GTCGCTTCCCAAATTACATC	CTCCATATCGTTCCAGTCG

Table S4. The primers used in RNAi experiments.

**Primer name**	**Primer sequence(5'-3') (T7=TAATACGACTCACTATAGGG)**
dsCsUGT40AL1-F	T7-ATTGGAAGCTAGTTGGGGGT
dsCsUGT40AL1-R	T7-GCTGGTTTCAAATCTTTCGC
dsCsUGT33AG3-F	T7-GAGGCAGCAAGAATATTGGC
dsCsUGT33AG3-R	T7-ACAGCAATGGATGGTTAGGC
dsEGFP-F	T7-AAGTTCAGCGTGTCCG
dsEGFP-R	T7-CACCTTGATGCCGTTC
